# Risk stratification based on change in plasma Epstein-Barr virus DNA load after treatment in nasopharyngeal carcinoma

**DOI:** 10.18632/oncotarget.7083

**Published:** 2016-01-30

**Authors:** Yuan Zhang, Wen-Fei Li, Yan-Ping Mao, Rui Guo, Ling-Long Tang, Hao Peng, Ying Sun, Qing Liu, Lei Chen, Jun Ma

**Affiliations:** ^1^ Department of Radiation Oncology, Sun Yat-Sen University Cancer Center, State Key Laboratory of Oncology in South China, Collaborative Innovation Center of Cancer Medicine, Guangzhou 510060, People's Republic of China; ^2^ Department of Cancer Prevention Research, Sun Yat-Sen University Cancer Center, State Key Laboratory of Oncology in South China, Collaborative Innovation Center of Cancer Medicine, Guangzhou 510060, People's Republic of China

**Keywords:** nasopharyngeal neoplasms, tumor markers, biological, Epstein-Barr virus, DNA

## Abstract

Background: Nasopharyngeal carcinoma is associated with Epstein-Barr virus (EBV). The current study investigated change in the plasma EBV DNA load in the first 3 months after treatment and its clinical significance in NPC.

Methods: A total of 273 patients with non-metastatic, histologically-proven NPC treated with radiotherapy or chemoradiotherapy were retrospectively reviewed.

Results: EBV DNA was detectable in 19/273 (7.0%) patients at the end of therapy (end-DNA). Three months later, 16/273 (5.9%) patients had detectable EBV DNA (3-month-DNA). To investigate risk stratified by the pattern of change in post-treatment EBV-DNA, we divided patients into four subgroups: Group 1, undetectable end-DNA and 3-month-DNA (*n* = 244); Group 2, detectable end-DNA and undetectable 3-month-DNA (*n* = 13); Group 3, undetectable end-DNA and detectable 3-month-DNA (*n* = 7); and Group 4, detectable end-DNA and 3-month-DNA (*n* = 2). Patients with delayed remission of EBV DNA after treatment (Group 2) had significantly poorer 3-year DFS (48.6% vs. 89.7%, *P* < 0.001), DMFS (48.6% vs. 94.6%, *P* < 0.001) and OS (91.7% vs. 97.5%, *P* < 0.001) than those with persistently undetectable EBV DNA post-treatment (Group 1). Five of the seven patients with re-emergent EBV DNA (Group 3) and both patients with persistent EBV DNA post-treatment (Group 4) developed disease failure.

Conclusion: Plasma EBV DNA load continues to change during the first 3 months after treatment. The pattern of change in EBV DNA load post-treatment could help identify patients with different prognoses.

## INTRODUCTION

Nasopharyngeal carcinoma (NPC) is endemic in China, where over 33000 new cases – representing 40% of cases worldwide – were diagnosed in 2012 [1]. Radiotherapy is the primary treatment modality for non-disseminated NPC [2, 3]. Chemoradiotherapy is recommended for loco-regionally advanced disease [2–4]. The revolution in radiation techniques from two-dimensional (2D) conventional radiotherapy to intensity-modulated radiotherapy (IMRT) has significantly improved local control, with a current local failure rate of 5–7% [5–7]. However, 15–21% of patients still develop distant metastasis after radical treatment, and distant metastasis has become the major failure pattern in NPC [5, 8].

NPC is associated with Epstein-Barr virus (EBV) infection. Many studies have demonstrated that EBV contributes to the pathogenesis of NPC [9–11], and EBV infection is ubiquitously detected in the primary and metastatic tumor cells of almost every patient with NPC, regardless of the geographic origin of the patient or the degree of tumor differentiation [11–17]. In patients with NPC, plasma EBV deoxyribonucleic acid (DNA) has been demonstrated to contain the same polymorphisms as the EBV DNA detected in tumor cells, which indicates that plasma EBV DNA originates from the tumor. Moreover, the plasma EBV DNA load correlates strongly with the tumor burden [18], thus providing a solid basis for the utility of assessing the plasma EBV DNA load in NPC.

To date, several studies have demonstrated that plasma EBV DNA represents a valuable tumor marker for the diagnosis, treatment response monitoring, prognostication and follow-up of patients with NPC [18–27]. A high pretreatment plasma EBV DNA load correlates with advanced stage and poor prognosis. Moreover, patients with detectable post-treatment EBV DNA have an extremely high risk of treatment failure [18, 19, 25–27]. However, the time-point of the post-treatment EBV DNA assessment in previous studies varies from within one week to three months after the end of treatment. Interestingly, at our clinic, we have observed that some patients with detectable EBV DNA at the end of treatment subsequently develop spontaneous remission of EBV DNA; however, no previous studies have addressed this issue. Thus, we aimed to assess the patterns of change in the EBV DNA load in the first 3 months after treatment and investigate its potential clinical significance in NPC.

## RESULTS

The median follow-up time was 38.4 months (range, 5.13–57.4 months). A total of 48 patients experienced failure: 10/273 (3.7%), 10/273 (3.7%), and 33/273 (12.1%) patients developed local recurrence, regional recurrence, and distant metastases, respectively; 18/273 (6.6%) patients died and 4/273 (1.5%) patients experienced both local-regional recurrence and distant metastases. The 3-year DFS, DMFS, LRRFS, and OS rates for the entire cohort were 83.4%, 88.5%, 93.5%, and 94.7%, respectively.

### Patterns of change in post-treatment plasma EBV DNA load

In total, EBV DNA was detectable in 19/273 (7.0%) patients at the end of therapy (end-DNA; median 0, range 0-78900 copies/ml). Three months later, 16/273 (5.9%) patients had detectable EBV DNA (3-month-DNA; median 0, range 0-2500000 copies/ml).

**Figure 1:**
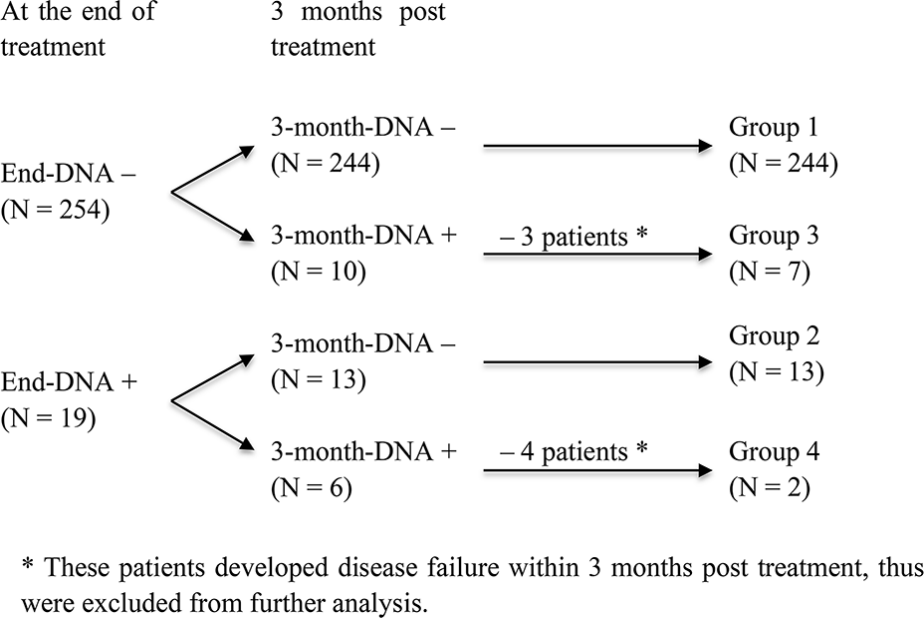
Flow chart of the study design

The objective of the current study was to investigate the prognostic value of end-DNA and 3-month-DNA for long-term survival. Thus, 7/273 patients who developed distant metastasis within 3 months after treatment were excluded from further analysis (Figure 1). The remaining 266 patients alive without disease at 3 months after treatment were included in the following analysis and their characteristics are shown in Table 1.

**Table 1:** Clinicopathological characteristics of the 266 patients with NPC

Characteristic	No. of 266 patients
**Sex**	
Male	196 (73.7%)
Female	70 (26.3%)
**Age (years)**	
≤ 50	192 (72.2%)
> 50	74 (27.8%)
**Histological type^a^**	
Non-keratinizing carcinoma	
Differentiated	12 (4.5%)
Undifferentiated	254 (95.5%)
**Pretreatment EBV DNA load**	
Median (*10^3^ copies/ml)	1.17
Range (*10^3^ copies/ml)	0–2470
**Chemotherapy**	
Yes	214 (80.5%)
No	52 (19.5%)
**T-category^b^**	
T1	64 (24.0%)
T2	43 (16.2%)
T3	117 (44.0%)
T4	42 (15.8%)
**N-category^b^**	
N0	52 (19.5%)
N1	146 (54.9%)
N2	52 (19.5%)
N3a	2 (0.8%)
N3b	14 (5.3%)
**Stage^b^**	
I	22 (8.3%)
II	61 (22.9%)
III	127 (47.7%)
IV	56 (21.1%)

Abbreviations: NPC, nasopharyngeal carcinoma; T, tumor; N, node.

a Pathologic type: according to the 2005 World Health Organization classification of tumors.

b According to the 7th edition of the UICC/AJCC staging system.

### Relationship between end-DNA/3-month-DNA load and clinical outcome

In univariate analysis, detectable end-DNA correlated with significantly poorer clinical outcomes in terms of DFS (41.7% vs. 88.0%, *P* < 0.001), DMFS (41.7% vs. 93.6%, *P* < 0.001) and OS (92.9% vs. 96.7%, *P* = 0.001), but not LRRFS (92.9% vs. 93.5%, *P* = 0.959). Moreover, detectable 3-month-DNA correlated with significantly poorer DFS (16.7% vs. 87.8%, *P* < 0.001), DMFS (38.1% vs. 92.5%, *P* < 0.001), OS (65.6% vs. 97.3%, *P* = 0.013) and LRRFS (62.2% vs. 94.4%, *P* = 0.001).

In multivariate analysis incorporating sex, age, T and N classification, pre-DNA, end-DNA, 3-month-DNA and chemotherapy as covariates, end-DNA was an independent prognostic factor for DFS (HR, 3.568; 95% CI, 1.569-8.116; *P* = 0.002), DMFS (HR, 6.967; 95% CI, 2.862-16.961; *P* < 0.001) and OS (HR, 8.739; 95% CI, 2.443-28.733; *P* = 0.001). Additionally, 3-month-DNA was an independent prognostic factor for DFS (HR, 5.979; 95% CI, 2.429-14.716; *P* < 0.001) and DMFS (HR, 4.974; 95% CI, 1.704-14.518; *P* = 0.003); detectable 3-month-DNA was also associated with an increased risk of death, though this trend did not reach statistical significance (HR, 4.772; 95% CI, 0.986-23.090; *P* = 0.052; Table 2).

**Table 2:** Summary of univariate and multivariate analyses of prognostic factors in the 266 patients with NPC

End-point	Variable	Univariate analysis	Multivariate analysis	
		*P*-value	HR (95% CI)	*P*-value
DFS	Pre-DNA	< 0.001	2.723 (1.300–5.704)	0.008
	End-DNA	< 0.001	3.568 (1.569–8.116)	0.002
	3-month-DNA	< 0.001	5.979 (2.429–14.716)	< 0.001
LRRFS	Gender	0.016	3.367 (1.318–8.603)	0.011
	3-month-DNA	< 0.001	9.749 (2.747–34.597)	< 0.001
DMFS	Pre-DNA	0.001	3.421 (1.250–9.366)	0.017
	End-DNA	< 0.001	6.967 (2.862–16.961)	< 0.001
	3-month-DNA	< 0.001	4.974 (1.704–14.518)	0.003
OS	T classification	0.058	4.609 (1.010–21.030)	0.049
	End-DNA	< 0.001	8.379 (2.443–28.733)	0.001
	3-month-DNA	0.004	4.772 (0.986–23.090)	0.052

Abbreviations: DFS, disease-free survival; LRRFS, local-regional recurrence-free survival; DMFS, distant metastasis-free survival; OS, overall survival.

### Risk stratification by pattern of change in post-treatment plasma EBV-DNA load

To investigate risk when stratified by the pattern of change in the post-treatment EBV-DNA load, we divided patients into four subgroups: Group 1, undetectable end-DNA and 3-month-DNA (*n* = 244); Group 2, detectable end-DNA and undetectable 3-month-DNA (*n* = 13); Group 3, undetectable end-DNA and detectable 3-month-DNA (*n* = 7); and Group 4, detectable end-DNA and 3-month-DNA (*n* = 2).

**Figure 2:**
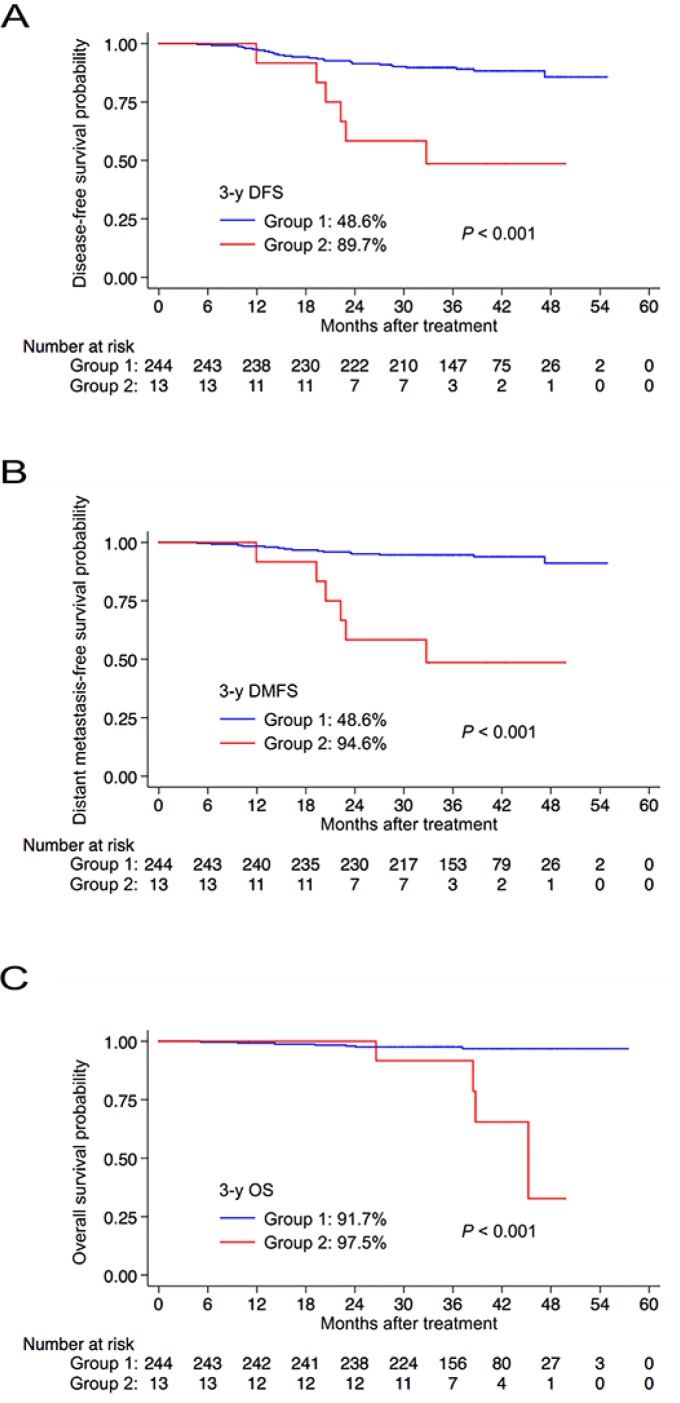
Kaplan-Meier curves for disease-free survival (**A**) distant metastasis-free survival (**B**) and overall survival (**C**) for patients in Group 1 and Group 2. Group 1 = patients with undetectable end-DNA and 3-month-DNA; Group 2 = patients with detectable end-DNA and undetectable 3-month-DNA. Abbreviations: 3-y = 3-year; DFS = disease-free survival; DMFS = distant metastasis-free survival; OS = overall survival.

For patients with delayed remission of EBV DNA (Group 2), the EBV DNA load at the end of treatment ranged from 31 to 18100 copies/ml (median, 165 copies/ml). Among the patients in Group 2, 10/13 (71.4%) had an end-DNA load < 500 copies/ml and 12/13 (85.7%) had an end-DNA load < 1000 copies/ml. Six of the 13 (46.2%) patients in Group 2 experienced disease failure; all six patients developed distant metastasis and one patient also developed local and regional recurrence. The Kaplan-Meier survival curves for Group 1 and Group 2 are shown in Figure 2. Patients who had delayed remission of EBV DNA after treatment had significantly poorer 3-year DFS (48.6% vs. 89.7%, *P* < 0.001), DMFS (48.6% vs. 94.6%, *P* < 0.001) and OS (91.7% vs. 97.5%, *P* < 0.001) than patients with persistently undetectable EBV DNA after treatment. Moreover, the pattern of change in EBV DNA load in these patients (Group 1 vs. Group 2) retained independent prognostic value for DFS (HR, 4.656; 95% CI, 1.909-11.354; *P* = 0.001), DMFS (HR, 8.785; 95% CI, 3.350-23.038; *P* < 0.001) and OS (HR, 11.568; 95% CI, 3.379-39.601; *P* < 0.001) in multivariate analyses.

Of the seven patients with undetectable end-DNA and detectable 3-month-DNA (Group 3), 5/7 developed disease failure at 3.2-27.5 months (median, 15.9 months) after the detection of plasma EBV DNA at 3 months post-treatment: two developed distant metastasis, two developed local-regional recurrence and one developed both distant metastasis and local-regional recurrence. The 3-month-DNA load of the five patients with disease failure ranged from 640 to 43500 copies/ml (median, 20800 copies/ml). However, the other two patients in Group 3 remained disease-free up to the last follow-up (follow up period: 30.6 and 26.0 months, respectively), with EBV DNA of 41 copies/ml and 194 copies/ml at 3 months and undetectable EBV DNA at subsequent follow-up visits. Detailed information for the seven patients in Group 3 is provided in Table 3.

**Table 3:** Clinical features of the seven patients with undetectable end-DNA and detectable 3-month-DNA (Group 3)

	Patient 1	Patient 2	Patient 3	Patient 4	Patient 5	Patient 6	Patient 7
Sex	Male	Male	Female	Male	Male	Male	Male
Age	77	49	48	40	75	37	49
Stage	T3N1M0, Stage III	T3N1M0, Stage III	T1N2M0, Stage III	T3N1M0, Stage III	T3N2M0, Stage III	T3N2M0, Stage III	T4N1M0, Stage IV
Chemotherapy^1^	N	C	C	I + C	N	C	I + C
Pre-EBV (copies/ml)	2330	9280	1460	21700	87300	7780	6320
3-month-EBV (copies/ml)	41	194	640	11900	26000	20800	43500
Disease failure site	No	No	Local	Distant	Distant	Regional	Regional +
							Distant
Time to disease failure^2^			22.8	13.1	9.9	3.1	3.0
Outcome	Alive	Alive	Alive	Alive	Dead	Alive	Dead
Follow-up time^3^	26.0	30.6	50.8	44.4	34.4	13.8	21.8

Abbreviations: *N* = no; *I* = induction chemotherapy; *C* = concurrent chemotherapy.

1 Patient 1 and patient 5 did not receive chemotherapy due to advanced age and comorbidity.

2 From the detection of abnormal plasma EBV DNA at 3 months post treatment.

3 From the first day of treatment.

Finally, EBV DNA was detectable at both the end of therapy and 3 months after therapy in two patients (Group 4). The pre-EBV, end-EBV and 3-month-EBV loads for these patients were 250000 and 2420 copies/ml, 393 and 17 copies/ml, and 186000 and 502 copies/ml, respectively. Both of the patients in Group 4 developed distant metastasis 15.3 months after treatment (Table 4).

**Table 4:** Clinical features of the two patients with detectable end-DNA and detectable 3-month-DNA (Group 4)

	Patient 1	Patient 2
Sex	Male	Male
Age	38	44
Stage	T2N3aM0 Stage IVA	T3N0M0 Stage III
Chemotherapy	I + C	C
Pre-EBV (copies/ml)	250000	24200
Post-EBV (copies/ml)	393	17
3-month-EBV (copies/ml)	186000	502
Disease failure	Distant metastasis	Distant metastasis
Sites of metastases	Mediastinal lymph nodes, bronchial lymph nodes	Bone, inguinal lymph nodes
Time to metastasis^1^	15.3 months	15.3 months
Outcome	Alive, with tumor	Alive, with tumor
Follow-up time^2^	46.3	26.8

Abbreviations: *N* = no; *I* = induction chemotherapy; *C* = concurrent chemotherapy.

1 From the end of treatment.

2 From the first day of treatment.

## DISCUSSION

In the current study, we provide the first report of spontaneous remission of EBV DNA in patients with detectable EBV DNA at the end of treatment. Patients with delayed remission of post-treatment EBV DNA, even those with a quite low EBV DNA load (median, 165 copies/ml) at the end of treatment, had a significantly poorer prognosis than patients with undetectable EBV DNA at both the end of therapy and 3 months after treatment. The mechanism underlying this phenomenon is unknown, but may possibly be related to a relatively poor sensitivity to radiotherapy and/or chemotherapy. On the other hand, two patients had detectable EBV DNA at the end of therapy and 3 months after treatment, and both of these patients developed distant metastasis. One possible explanation is that detectable post-treatment EBV DNA reflects the presence of residual tumor cells [19], which may lead to disease failure. Thus, patients with detectable EBV DNA at the end of therapy are at extremely high risk of disease failure-and despite the fact that spontaneous remission of EBV DNA may occur without further interventions-these patients may benefit from additional adjuvant chemotherapy [28].

Meanwhile, plasma EBV DNA was undetectable at the end of treatment but reemerged 3 months later in seven patients, of whom five subsequently developed disease failure. Further studies are warranted to investigate the biological foundation of this phenomenon. In these five patients, the reemergence of EBV DNA preceded the clinical signs of disease failure. As the effectiveness of salvage treatment is closely related to the tumor burden at the time of relapse [29–32], close monitoring of EBV DNA after treatment may enable the early detection of treatment failure and improve the outcome of salvage therapy.

To date, several studies have reported that patients with detectable post-treatment EBV DNA have a significantly poorer prognosis [18, 19, 25–27]. However, the rates of detectable post-treatment EBV DNA in previous reports varied significantly from 10% to 28.8%, possibly due to varied EBV DNA assessment time-points ranging from within one week to three months after treatment. For instance, Leung et al. reported that 16% of patients had detectable EBV DNA within 3 months of the completion of therapy. In this cohort of patients, the rate of residual EBV DNA at the end of therapy (7%) was lower than previous reports, possibly due to that the majority of patients in previous studies received 2D conventional radiotherapy, while all patients in our cohort received IMRT. Here, we demonstrate that the plasma EBV DNA load continues to change during the first 3 months after treatment. Our results suggest that routine EBV DNA assays should be performed at the end of treatment and subsequent follow-up visits; this may provide important information about the patients’ outcome. Additionally, in future studies involving post-treatment EBV DNA, it would be better to define a uniform time-point for assessing EBV DNA, especially in multicenter studies, to reduce potential bias. Major cooperation groups including the NRG are currently conducting several multi-institutional clinical trials in order to improve the outcome of patients with detectable post-treatment EBV DNA. However, the NRG HN001 (NCT02135042) and National Health Research Institute in Taiwan study (NCT02363400) assesses post-treatment EBV DNA within 1 week after treatment, while the Hong Kong Nasopharyngeal Cancer Study Group trial (NCT00370890) assesses EBV DNA at 6-8 weeks after treatment, which will lead to difficulties when comparing the results of these trials. The optimal time-points for assessing the residual post-treatment EBV DNA load to guide further intervention needs to be addressed in the future.

This study has some limitations. Firstly, biases due to the retrospective nature of the analyses are unavoidable, and no validation dataset was included. However, the data in the present study are consistent with-and also provide a new perspective on-previous studies on the prognostic value of post-treatment EBV DNA. Secondly, there were limited numbers of patients in Group 3 and Group 4 (nine patients). However, almost all of the patients in these groups (7/9) developed disease failure, which clearly indicates the extremely high risk of disease failure in these patients.

In conclusion, the plasma EBV DNA load of patients with NPC continues to change during the first 3 months after treatment. The pattern of change in the post-treatment EBV DNA load could help to identify patients with different prognoses. Future clinical trials are warranted to tailor individualized treatment based on post-treatment EBV DNA.

## PATIENTS AND METHODS

### Patients

This study was approved by the institutional review board; requirement to obtain informed consent was waived. Between January 2010 and December 2011, 273 patients with non-metastatic NPC treated at our institution received an EBV DNA assay before, at the end of (+/− 1 week) and 3 months (+/− 1 week; i.e. at the first follow-up visit) after treatment. All of these patients received intensity-modulated radiotherapy (IMRT) with or without chemotherapy. The median age of the remaining 273 patients was 44.4 years (range, 14–77 years), with a male-to-female ratio of 2.8:1.

All patients underwent a pretreatment evaluation including a complete patient history, physical examination, hematology and biochemistry profiles, MRI of the neck and nasopharynx, chest radiography, abdominal sonography, and whole body bone scan using single photon emission computed tomography (SPECT). Furthermore, positron emission tomography-computed tomography (PET-CT) was performed on 123/273 (45.0%) patients. All patients were staged according to the 7th edition of the International Union against Cancer/American Joint Committee on Cancer (UICC/AJCC) system [33].

### DNA extraction and real-time quantitative polymerase chain reaction analysis

Plasma EBV DNA load was detected before the start of treatment, at the end of treatment (+/− 1 week) and 3 months after the completion of treatment (+/− 1 week). Samples of peripheral blood (3 ml) were collected into ethylenediamine tetraacetic acid (EDTA) tubes and subjected to centrifugation at 16000 g for 5 min to isolate plasma. The plasma was transferred to fresh tubes and stored at −80°C. DNA was isolated from 500 μl aliquots of plasma using the QIAamp DNA Blood MiniKit (Qiagen, Hilden, Germany), according to the manufacturer's instructions.

The plasma EBV DNA concentration was measured using a real-time quantitative PCR assay which targets the BamH I-W region of the EBV genome, as previously described [34]. The primer sequences were: 5′-GCCAG AGGTA AGTGG ACTTT-3′ (F) and 5′-TACCA CCTCC TCTTCTTGCT-3′ (R). A dual fluorescence-labeled oligomer, 5′ (FAM) CACAC CCAGG CACAC ACTAC ACAT (TAMRA) 3′, served as a probe. Sequence data for the EBV genome was obtained from the GenBank sequence database. The plasma concentration of EBV DNA was calculated using the following equation: C = Q × (V_DNA_/V_PCR_) × (1/V_EXT_), in which C represents the target concentration in plasma (copies/ml), Q represents the target quantity (copies) determined by a sequence detector in a PCR reaction, V_DNA_ represents the total volume of DNA, V_PCR_ represents the volume of DNA solution used for PCR (typically 2 μl), and V_EXT_ represents the volume of plasma extracted (typically 0.5 ml) [34].

### Treatment

The nasopharyngeal and neck tumor volumes of all patients were treated using radical radiotherapy based on IMRT for the entire treatment course. All targets were treated simultaneously using the simultaneous integrated boost technique; other details of the techniques used at our center have been reported previously [35].

During the study, institutional guidelines recommended radiotherapy only for stage I and concurrent chemoradiotherapy ± neoadjuvant/adjuvant chemotherapy for stage II to IVB NPC. In total, 92.7% (177/191) of patients with stage III-IVB disease received concurrent chemoradiotherapy ± neoadjuvant/adjuvant chemotherapy. When possible, salvage treatments (intracavitary brachytherapy, surgery or chemotherapy) were provided in documented persistent disease or relapse.

### Follow-up

Patients were examined at least every 3 months during the first 2 years, and every 6 months during years 3–5 or until death. Evaluation during follow-up included a complete patient history, physical examination, hematology and biochemistry profiles, MRI of the neck and nasopharynx, chest radiography, abdominal sonography and a whole body bone scan. All local recurrences were diagnosed via fiber-optic endoscopy and biopsy, a MRI scan of the nasopharynx and the skull base showing progressive bone erosion or soft tissue swelling, or both. Regional recurrences were diagnosed by clinical examination of the neck and, in doubtful cases, by fine needle aspiration or a MRI scan of the neck. Distant metastases were diagnosed by clinical symptoms, physical examinations, and imaging methods that included chest radiography, bone scan, MRI, CT, and abdominal sonography [36].

### Statistical analysis

Statistical analyses were performed using SPSS version 22.0 (IBM Corporation, Armonk, NY, USA). The following endpoints were assessed: disease-free survival (DFS), distant metastasis-free survival (DMFS), local-regional recurrence-free survival (LRRFS) and overall survival (OS). DMFS, LRRFS and OS were calculated from the first day of treatment to the first distant metastasis, local-regional relapse or death, respectively. DFS was defined as the latency to the date of disease progression or death from any cause. Survival rates were calculated using the Kaplan-Meier method and compared using the log-rank test [37]. Multivariate analyses with the Cox proportional hazards model were used to calculate HRs, 95% confidence intervals (CIs), and to test the independent significance of different factors by backward elimination of insignificant variables [38], and included host factors (sex, age), therapeutic intervention (chemotherapy), pretreatment plasma EBV DNA load (pre-DNA < median vs. pre-DNA > median) and tumor factors (T classification; N classification) as covariates. Two-tailed *P*-values < 0.05 were considered statistically significant.
